# Supplementary material for: Transcriptomic Insights into Mechanisms of Early Seed Maturation in the Garden Pea (Pisum sativum L.)
Source: Cells. 2020 Mar 23;9(3):779. doi: 10.3390/cells9030779 (PMC7140803; doi:10.3390/cells9030779)

Zhongwan−6,10(1)

Global Summary

%DE = 0.23

genes with fdr < 0.2 = 468 (216 + / 252 −)

genes with fdr < 0.1 = 432 (197 + / 235 −)

genes with fdr < 0.05 = 386 (178 + / 208 −)

genes with fdr < 0.01 = 328 (150 + / 178 −)

genes in genesets = 0

<FC> = 0

<t−score> = 0

<p−value> = 0.01

<fdr> = 0.77

Global Genelist

Rank	ID	log(FC)	p−value	fdr	Metagene	Description
1	TRINITY_DN1	−1.32	2e−16	3e−15	30 x 1	
2	TRINITY_DN2	2.56	2e−16	3e−15	1 x 1	
3	TRINITY_DN1	1.69	2e−16	3e−15	1 x 1	
4	TRINITY_DN9	−0.72	2e−16	3e−15	30 x 30	
5	TRINITY_DN7	2.02	2e−16	3e−15	30 x 1	
6	TRINITY_DN7	−3.66	2e−16	3e−15	30 x 30	
7	TRINITY_DN5	−2.16	2e−16	3e−15	30 x 30	
8	TRINITY_DN5	0.98	2e−16	3e−15	1 x 1	
9	TRINITY_DN7	1.41	2e−16	3e−15	2 x 1	
10	TRINITY_DN7	3.33	2e−16	3e−15	1 x 1	
11	TRINITY_DN9	0.73	2e−16	3e−15	5 x 6	
12	TRINITY_DN4	5.77	2e−16	3e−15	1 x 1	
13	TRINITY_DN9	1.92	2e−16	3e−15	1 x 1	
14	TRINITY_DN2	−1.6	2e−16	3e−15	1 x 30	
15	TRINITY_DN2	2.07	2e−16	3e−15	1 x 1	
16	TRINITY_DN2	−1.09	2e−16	3e−15	30 x 1	
17	TRINITY_DN2	−1.62	2e−16	3e−15	30 x 30	
18	TRINITY_DN2	1.06	2e−16	3e−15	1 x 1	
19	TRINITY_DN2	−0.76	2e−16	3e−15	30 x 30	
20	TRINITY_DN2	2.25	2e−16	3e−15	1 x 1	

Portrait

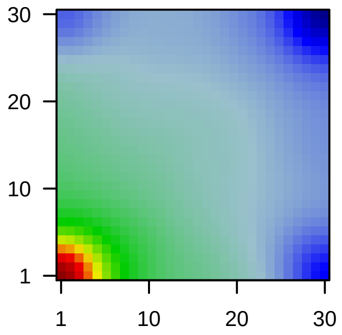

Top 100 DE genes

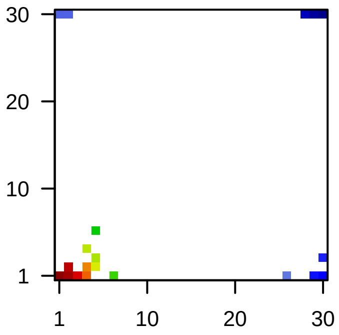

p−values

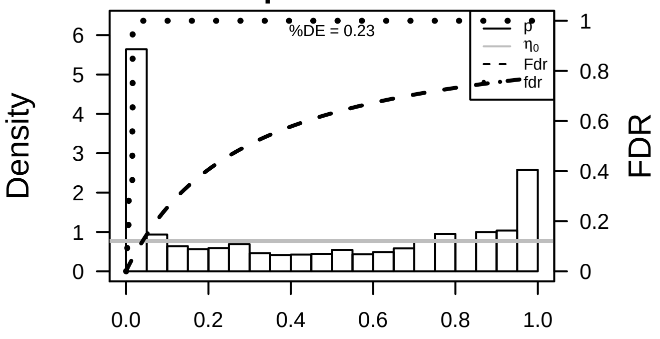

Supplement: Supplementary file 1 [file cells-09-00779-s001.zip › Supplementary materials/FileS4.zip!/FileS3/TFs - Results/Summary Sheets - Samples/Zhongwan.6.10.1..pdf]
